# The Etiology of Syphilis

**Published:** 1905-06-10

**Authors:** 


### THE ETIOLOGY OF SYPHILIS.

MM. Roux and Metchnikoff,[1] of the Pasteur Institute in Paris, have supported with their authority the discovery by Schaudinn, of Berlin, of a spirochæte constantly associated with the early lesions of syphilis, and very probably the causal organism of this disease. The name Spirochæte pallida has been given to this organism, which is small and of a corkscrew shape, and hard to stain. Schaudinn and his fellow-workers have examined 26 primary sores and mucous patches, and have always found this spirochæte, though sometimes with difficulty. They have found it not only in the discharges of the lesions, but also in the deeper layers of the infected tissues, and in the inguinal glands affected during the primary and secondary stages. MM. Roux and Metchnikoff have examined six of the anthropoid apes recently infected at the Institute, and obtained positive results in four of them, one of the failures being an ape on the road to recovery after treatment by an anti-syphilitic serum. They subsequently examined scrapings from the secondary papules in human subjects, and obtained positive results in four out of six examinations. The organism was most easily found in very young papules, which reduces the likelihood of its being a skin-contamination; further, all attempts to recover the spirochæte pallida from non-syphilitic skin lesions such as psoriasis, acne and scabies, have so far failed. As no success has hitherto attended efforts to grow other spirilla such as that which causes relapsing fever, it is not surprising to learn that the new organism has evaded cultivation; but anti-syphilitic sera are being prepared by the use of virulent material obtained from primary and secondary lesions. Until success in cultivation is achieved, some doubt must unfortunately remain as to the actual relation between the organism and the disease, but there is strong evidence that the mystery of syphilis is at last yielding to scientific effort.

[1] Brit. Med. Jour., May 27, 1905.

